# Amphiregulin enhances alpha6beta1 integrin expression and cell motility in human chondrosarcoma cells through Ras/Raf/MEK/ERK/AP-1 pathway

**DOI:** 10.18632/oncotarget.3397

**Published:** 2015-03-18

**Authors:** Jui-Chieh Chen, Yu-Ju Chen, Chih-Yang Lin, Yi-Chin Fong, Chin-Jung Hsu, Chun-Hao Tsai, Jen-Liang Su, Chih-Hsin Tang

**Affiliations:** ^1^ Department of Biochemical Science and Technology, National Chiayi University, Chiayi, Taiwan; ^2^ Graduate Institute of Basic Medical Science, China Medical University, Taichung, Taiwan; ^3^ National Institute of Cancer Research, National Health Research Institutes, Miaoli County, Taiwan; ^4^ School of Pharmacy, China Medical University, Taichung, Taiwan; ^5^ School of Chinese Medicine, College of Chinese Medicine, China Medical University, Taichung, Taiwan; ^6^ Department of Orthopedic Surgery, China Medical University Hospital, Taichung, Taiwan; ^7^ Department of Medicine and Graduate Institute of Clinical Medical Science, China Medical University, Taichung, Taiwan; ^8^ Department of Biotechnology, College of Health Science, Asia University, Taichung, Taiwan; ^9^ Center for Molecular Medicine, China Medical University Hospital, Taichung, Taiwan; ^10^ Graduate Institute of Cancer Biology, China Medical University, Taichung, Taiwan; ^11^ Department of Pharmacology, School of Medicine, China Medical University, Taichung, Taiwan

**Keywords:** Chondrosarcoma, Amphiregulin, Integrin, Cell migration

## Abstract

Chondrosarcoma is a malignant tumor that produces cartilage matrix. The most lethal aspect is its metastatic property. We demonstrated that amphiregulin (AR) is significantly upregulated in highly aggressive cells. AR silencing markedly suppressed cell migration. Exogenous AR markedly increased cell migration by transactivation of α6β1 integrin expression. A neutralizing α6β1 integrin antibody can abolish AR-induced cell motility. Knockdown of AR inhibits metastasis of cells to the lung *in vivo*. Furthermore, elevated AR expression is positively correlated with α6β1 integrin levels and higher grades in patients. These findings can potentially serve as biomarker and therapeutic approach for controlling chondrosarcoma metastasis.

## INTRODUCTION

Chondrosarcoma, the second most common type of bone cancer, is a heterogeneous group of malignancies that are characterized by the production of cartilage matrix. High-grade chondrosarcoma is more aggressive and is more likely to metastasize to other areas of the body. Although high-grade only occurs in approximately 5–10% of chondrosarcoma patients, it remains the major cause of death [1]. Therefore, metastasis is a major obstacle that must be overcome for the successful treatment of chondrosarcoma. Exploring the molecular basis of metastasis may provide further improvements in early detection, prevention, intervention, and prognostic evaluation for patients with chondrosarcoma.

Secreted proteins are responsible for the cross talking among cells, which may facilitate the progression of metastasis, particularly within the steps of migration and invasion [2]. Amphiregulin (AR), a ligand of the epidermal growth factor receptor (EGFR), is synthesized as a transmembrane precursor that undergoes a series of proteolytic process to yield mature secreted form [3]. In normal bone development, AR exerts their biological function by mediating cell migration [4, 5]. AR has also emerged as an important predictive marker for metastasis in cancer [6, 7]. Accumulating evidence reveals that high-level expression of AR is associated with cancer progression in various types of cancers [8–14].

Previous studies have demonstrated that EGF and AR are capable of promoting cell motility by increasing integrin expression [15–17]. AR knock-down cells revealed a dramatic decrease in invasive capability and microarray data indicated a statistically significant difference in integrin expression [18]. Integrins are a family of heterodimeric transmembrane glycoproteins and have been shown to mediate cell-cell or cell-matrix interactions. To date, at least 24 unique integrin heterodimers have been identified. These heterodimers are formed from various combinations of 18 α-subunits and 8 β-subunits by non-covalent interactions, which play a crucial role of metastasis in the cancer biology [19–21].

Although the roles of AR have emerged as a pivotal factor in the regulation of cell motility across diverse cancer, the effect of AR on migration of chondrosarcoma cells still remains largely unknown. In the present study, we probed intracellular signal pathways involved in AR-induced integrin expression to regulate cell migration in human chondrosarcoma cells.

## RESULTS

### AR-induced cell migration through up-regulation of α6β1 integrin

**Figure 1:**
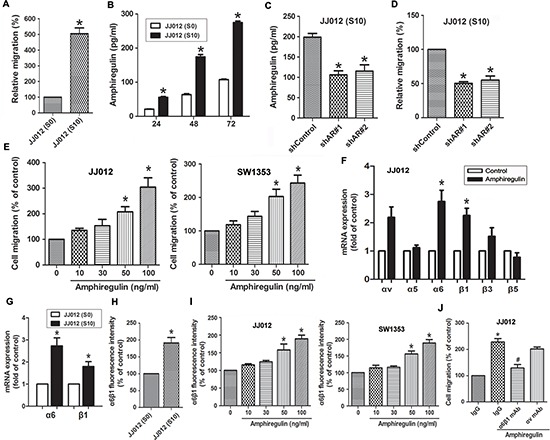
The expression level of AR is involved in cell migration and up-regulation of α6β1 integrin **A.** Relative cell migration between parental JJ012 (S0) and migration-prone subline JJ012 (S10) cells was determined by Transwell migration assays. **B.** Comparison of AR secretion between JJ012 (S0) and JJ012 (S10) was evaluated by ELISA. Serum-free supernatants of cell cultures were harvested at 24, 48, and 72 h. **C.** The protein levels of AR in JJ012 (S10)/shControl and JJ012 (S10)/shAR cells were examined by ELISA. **D.** AR knockdown suppressed the cell migration of JJ012 (S10) **E.** Cells were incubated with various concentrations of AR for 24 h. The effect of AR on cell migration was examined by Transwell assay (Left panels: JJ012 cells; Right panels: SW1353 cells). **F.** Cells were incubated with AR (50 ng/ml) for 24 h, and the mRNA levels of αv, α5, α6, β1, β3, or β5 integrin was determined using qPCR. **G.** The mRNA levels of α6β1 in JJ012 (S0) and JJ012 (S10) were examined by qPCR. **H.** The protein levels of α6β1 in JJ012 (S0) and JJ012 (S10) were examined by flow cytometry. **I.** Cells were incubated with various concentrations of AR for 24 h, and the cell surface expression of α6β1 integrin was determined using flow cytometry. **J.** Cells were pretreated with α6β1 or αv monoclonal antibody (10 μg/ml) for 30 min followed by stimulation with AR (50 ng/ml). The *in vitro* migration activity measured after 24 h. Results are expressed as mean ± SEM. *P < 0.05 compared with control; #*P* < 0.05 compared with AR-treated group.

To investigate whether AR is associated with migration activity in human chondrosarcoma cells, we first compared migration ability and the levels of AR secretion between JJ012 (S0) and JJ012 (S10). As shown in Fig. 1A, JJ012 (S10) cells had higher cell mobility and migrated more easily to the underside of the Transwell filters than did JJ012 (S0). Additionally, the levels of secreted AR protein were significantly higher in JJ012 (S10) compared to JJ012 (S0) cells, as assessed by ELISA (Fig. 1B). To clarify whether AR expression is involved in migration of chondrosarcoma cells, we knocked down AR expression by lentivirus-mediated delivery of AR shRNA. Knockdown efficiency of AR was determined by ELISA (Fig. 1C). As shown in Fig. 1D, silencing AR expression resulted in decreased migration of JJ012 (S10) cells. In addition to knockdown approach, we treated JJ012 (S0) cells with exogenous AR to assess its effect on cell migration. The results showed that AR enhanced cell migration ability in a concentration-dependent manner (Fig. 1E, left panel). This enhanced migration was also observed with SW1353 (another chondrosarcoma cell line) under the same conditions (Fig. 1E, right panel). In human chondrosarcomas, numerous studies have shown that increased integrin expression and signaling are implicated in cancer cell migration, invasion, and metastasis [1]. We therefore hypothesized that AR may promote cell migration by increasing the expression of specific integrins. The q-PCR analysis showed that AR induced α6 and β1 but not αv, α5, β3, and β5 integrin expression (Fig. 1F). To clarify whether JJ012 (S10) express higher level of α6β1 integrin than did JJ012 (S0), we examined the α6β1 integrin level by qPCR and flow cytometry. The results indicated that α6β1 integrin expressed level in JJ012 (S10), at both mRNA and protein levels, was drastically higher than JJ012 (S0) (Fig. 1G–1H). In addition, AR-induced α6β1 integrin expression was further validated by flow cytometry in different chondrosarcoma cells. As shown in the left panel of Fig. 1I, treatment of JJ012 cells with AR induced the cell surface expression of α6β1 integrin. Similar results were obtained in SW1353 cells (Fig. 1I, right panel). To further confirm the effect of AR on migration through α6β1 integrin, cells pre-treated with anti-α6β1 monoclonal antibody markedly inhibited the AR-induced cell migration (Fig. 1J). However, no significant inhibitory effect was observed with the anti-αv monoclonal antibody (Fig. 1J).

### AR induces α6β1 expression through the Ras/Raf-1/MEK1/ERK pathway

**Figure 2:**
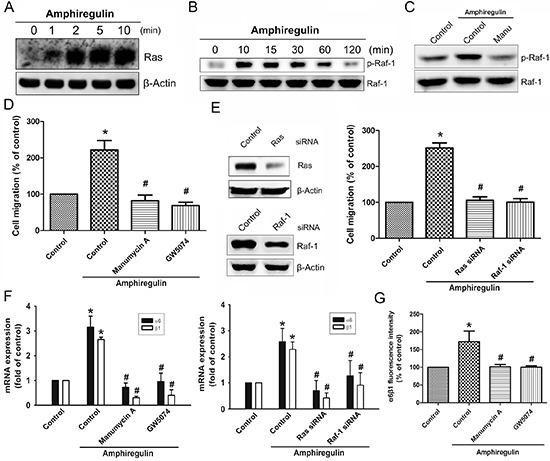
AR increased cell migration and α6β1 integrin expression via Ras and Raf-1 pathways Cells were incubated with AR (50 ng/ml) for the indicated time intervals. **A.** Ras activation was determined by pull-down binding to GST-Raf-1-RBD and subsequent immunoblotting with anti-Ras mAb. **B.** Phosphorylation of Raf-1 was determined by Western blot. **C.** Cells were pretreated with the manumycin A (10 μM) for 30 min, followed by treatment with AR (50 ng/ml) for 10 min. Phosphorylation of Raf-1 was analyzed by Western blot. **D.** Cells were pretreated with the manumycin A (10 μM) or GW5074 (10 μM) for 30 min, followed by treatment with AR (50 ng/ml) for 24 h. Cell migration was analyzed by Transwell assays. **E.** Cells were transfected with Ras and Raf-1 siRNA for 24 h, and then stimulated with AR (50 ng/ml) for 24 h. The knockdown efficiency of siRNA was verified by Western blot. The effect of knockdown on cell migration was examined by Transwell. **F.** Cells were pretreated with or without manumycin A or GW5074 for 30 min, or transfected with Ras siRNA or Raf-1 siRNA for 24 h followed by stimulation with AR (50 ng/ml). The mRNA expression level of α6β1 was examined by q-PCR. **G.** The protein expression levels of α6β1 integrin were examined by flow cytometry analysis. Results are expressed as mean ± SEM. *P < 0.05 compared with control; #*P* < 0.05 compared with AR-treated group.

**Figure 3:**
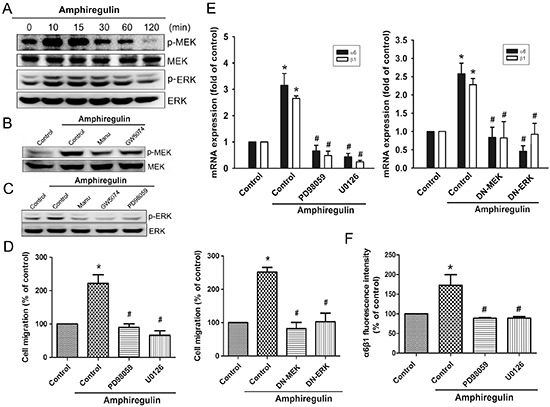
MEK and ERK pathways are involved in AR-induced increase in cell migration and α6β1 integrin expression **A.** Cells were incubated with AR (50 ng/ml) for indicated time intervals, p-MEK and p-ERK expression were determined by Western blot. **B.** Cells were pretreated with manumycin A or GW5074 for 30 min followed by stimulation with AR (50 ng/ml), and then p-MEK expression was examined by Western blot. **C.** Cells were pretreated with manumycin A, GW5074, or PD98059 for 30 min followed by stimulation with AR (50 ng/ml), and then p-ERK expression was examined by Western blot. **D-E.** Cells were pretreated with PD98059 (10 μM) and U0126 (10 μM) for 30 min or transfected with MEK1 and ERK mutant for 24 h followed by stimulation with AR (50 ng/ml) for 24 h, and *in vitro* migration and α6β1 integrin expression were analyzed by the Transwell and q-PCR, respectively. **F.** Cells were pretreated with PD98059 or U0126 for 30 min followed by stimulation with AR (50 ng/ml) for 24 h, and the protein levels of α6β1 integrin were determined by flow cytometry analysis. Results are expressed as mean ± SEM. *P < 0.05 compared with control; #*P* < 0.05 compared with AR-treated group.

To examine the mechanism by which AR induces α6β1 expression, we directly measured the Ras activity and Raf-1 phosphorylation in response to AR. The results revealed that stimulation of cells to AR induced an increase in Ras activity and phosphorylation of Raf-1 in a time-dependent fashion (Fig. 2A–2B). Pretreatment of cells with the Ras inhibitor attenuated phosphorylation of Raf-1, suggesting that Ras serves as upstream regulator of Raf-1-mediated signaling (Fig. 2C). Furthermore, AR-induced cell migration was significantly reduced by inhibition of Ras/Raf-1 signaling using either specific inhibitors or siRNAs (Fig. 2D–2E). Knockdown efficiency of Ras or Raf-1 was determined by Western blot (Fig. 2E, left). To examine whether AR stimulates the expression of α6β1 integrin via Ras/Raf-1 signaling, cells were blocked the pathway by either specific inhibitors or siRNAs. As shown in Fig. 2F, AR-induced expression of α6β1 integrin at the mRNA levels were strongly reduced in the presence of inhibitors or siRNA against Ras and Raf-1. Pretreatment of cells with manumycin A or GW5074 antagonized AR-induced expression of α6β1 integrin at the protein levels, as assessed by flow cytometry (Fig. 2G). Next, we investigated whether AR is able to activate MEK/ERK that is a critical downstream target of Raf-1. Stimulation of cells with AR induced a time-dependent phosphorylation of MEK and ERK (Fig. 3A). However, AR-induced phosphorylation of MEK/ERK was markedly decreased by inhibiting upstream signaling events using pharmacological inhibitors (Fig. 3B–3C). To further evaluate the MEK1/ERK pathway is able to induce the cell migration and α6β1 integrin expression, we pretreated cells with PD98059 (10 μM) and U0126 (10 μM), or transfected them with MEK1 and ERK mutant. As shown in Fig. 3D–3E, AR-induced cell migration and α6β1 integrin expression were greatly reduced when the MEK/ERK pathway was inactivated. Furthermore, AR-induced the protein levels of α6β1 integrin were also significantly abolished when pretreated cells with PD98059 and U0126 (Fig. 3F).

### Transcription factor AP-1 is required for AR-mediated α6β1 integrin in human chondrosarcoma cells and subsequently elicit cell migration

**Figure 4:**
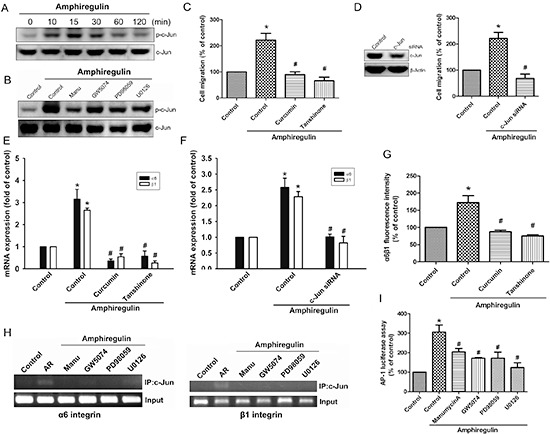
Activation of c-Jun is required for AR-induced cell migration and up-regulation of integrin α6β1 **A.** Cells were incubated with AR (50 ng/ml) for indicated time intervals. The phosphorylation status and total levels of c-Jun were measured by Western blot. **B.** Cells pretreated with manumycin A, GW5074, PD98059, or U0126 for 30 min followed by treatment with AR (50 ng/ml) for 15 min. The levels of p-c-Jun and c-Jun were measured by Western blot. **C-G.** Cells were pretreated with curcumin (10 μM) or tanshinone (10 μM) for 30 min or transfected with c-Jun siRNA for 24 h followed by stimulation with AR (50 ng/ml) for 24 h. The knockdown efficiency was verified by Western blot. The *in vitro* migration was measured by Transwell assay (C-D) The expression of integrin α6β1 was measured by q-PCR (E-F) and flow cytometry (G). **H.** Cells were pretreated with manumycin A, GW5074, PD98059, or U0126 for 30 min, followed by stimulation with AR (50 ng/ml), and chromatin immunoprecipitation assay was then performed. Chromatin was immunoprecipitated with anti-c-Jun antibody. One percent of the precipitated chromatin was assayed to verify equal loading (input). **I.** Cells pretreated with manumycin A, GW5074, PD98059, or U0126, followed by stimulation with AR (50 ng/ml) for 24 h. Equal amounts of cell extract were assayed for dual-luciferase activity. Results are expressed as mean ± SEM. *P < 0.05 compared with control; #*P* < 0.05 compared with AR-treated group.

Previously studies have indicated that c-Jun, a component of AP-1 (activating protein-1), can be phosphorylated by MAPKs, leading to a significant increased in the activity of AP-1 [22–24]. We therefore hypothesized that AP-1 may be involved in AR-mediated expression of α6β1 integrin in human chondrosarcoma cells. Our data demonstrated that AR induced a significant increase in c-Jun phosphorylation (Fig. 4A), but this effect was attenuated by manumycin A, GW5074, PD98059, and U0126 (Fig. 4B). To further evaluate the activation of AP-1 is required for AR-induced migration, cells were pretreated with AP-1 inhibitors (curcumin and tanshinone) or transiently transfected with c-Jun siRNA, followed by stimulation with AR, and *in vitro* migration was measured by Transwell assay. The results revealed that AR elicited a significant rise in cell migration, which were drastically attenuated in the presence of AP-1 inhibitors (Fig. 4C) or knockdown of c-Jun (Fig. 4D). Knockdown efficiency of c-Jun was verified by Western blot (Fig. 4D, left). Similarly, inhibition of AP-1 activation, by chemical inhibitors or transfection of cells with a specific siRNA attenuated AR-induced expression of integrin α6β1 at mRNA levels (Fig. 4E–4F). Moreover, AP-1 inhibitors also markedly inhibited AR-induced the protein levels of integrin α6β1 expression (Fig. 4G). We next explored whether AR activates Ras/Raf-1/MEK1/ERK pathway, which then results in transcriptional activation of integrin α6β1 through binding to the functional AP-1 site. The *in vivo* recruitment of c-Jun to the promoter of integrin α6β1 was assessed by ChIP assay. The results demonstrated that AR significantly increased c-Jun binding to the AP-1 element of the α6 or β1 integrin promoter, but this binding was attenuated by manumycin A, GW5074, PD98059, and U0126 (Fig. 4H). To further confirm that the Ras/Raf-1/MEK1/ERK pathway signaling pathway involved in AR-induced AP-1 activation, we performed promoter activity assays using transient transfection with AP-1 promoter luciferase construct into cells. As shown in Fig. 4I, treatment of cells with AR caused an increase in AP-1-luciferase activity; however, pretreatment of cells with manumycin A, GW5074, PD98059, and U0126 antagonized AR-induced AP-1-luciferase activity. Taken together, these data suggest that activation of Ras, Raf-1, MEK1, and ERK are required for AR-induced AP-1 activation in human chondrosarcoma cells.

### Knockdown of AR inhibits metastasis of chondrosarcoma cells to the lung in animal models

**Figure 5:**
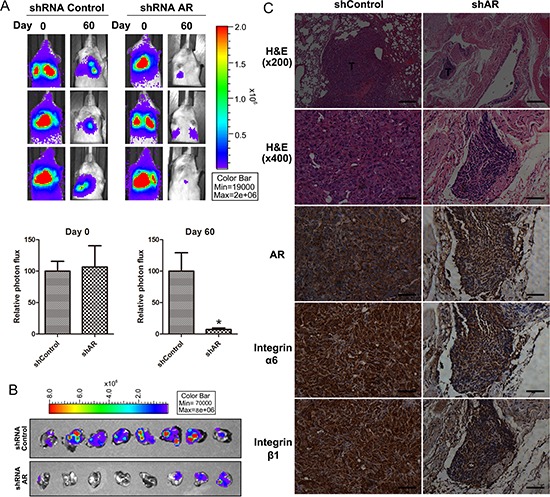
Depletion of AR suppresses metastasis of chondrosarcoma cells to the lung in mouse model **A.** Representative bioluminescent imaging of SCID mice injected via tail vein with JJ012 (S10)-Luc cells stably expressing control shRNA or AR shRNA. Color scale depicts the photon flux (photons/s) emitted from these mice. Whole-body bioluminescence imaging was performed at day 0 and 60 after intravenous inoculation. Quantification of bioluminescent imaging data is shown on the bottom (*n* = 8). **B.** Bioluminescent imaging of the lungs dissected from SCID mice was performed at the end of the experimental period. **C.** Representative images of lung tissue sections from mice injected with JJ012 (S10)-Luc cells with or without AR knockdown. Paraffin-embedded sections were stained with hematoxylin-eosin (H&E) or used for immunolabeling with antibodies against AR, integrin α6, and integrin β1. T indicates tumor metastases. Images were captured at 200x and 400x magnification as indicated. The scale bar for 200x is 200 μm and 400x is 50 μm.

To further investigate whether expression of AR would affect tumor metastasis *in vivo*, we monitored the metastatic potential of JJ012 (S10)-Luc cells stably expressing control shRNA or AR shRNA in mouse models of lung metastasis by using bioluminescence imaging. At day 0 post-injection, there were no differences between the control shRNA and AR shRNA groups, suggesting that mice were injected with the same number of cells. However, at day 60 after injection, tumor signals in the lung were significantly reduced when AR expression was knocked down (Fig. 5A). To confirm metastasis of tumor cells into lungs, we isolated lung tissues and took ex-vivo images (Fig. 5B). Next, we performed histological analyses of lung tissues from mice using hematoxylin-eosin (H&E) or immunohistological (IHC) staining. The results suggested that lung tissues from mice injected with AR knockdown JJ012 (S10)-Luc cells showed nearly normal structure of lungs or dramatically reduced the degree of lung metastatic nodules. However, lung tissues from control group mice were heavily infiltrated (Fig. 5C, H&E staining). Furthermore, an IHC analysis of the lungs revealed knockdown of AR led to lower expression of α6β1 integrin in metastatic nodules (Fig. 5C). Therefore, our results suggested that AR is involved in up-regulation of α6β1 integrin expression, which in turn promotes cancer metastasis to the lung.

### AR and α6β1 integrin expression levels positively correlate with the degree of malignancy in chondrosarcoma

**Figure 6:**
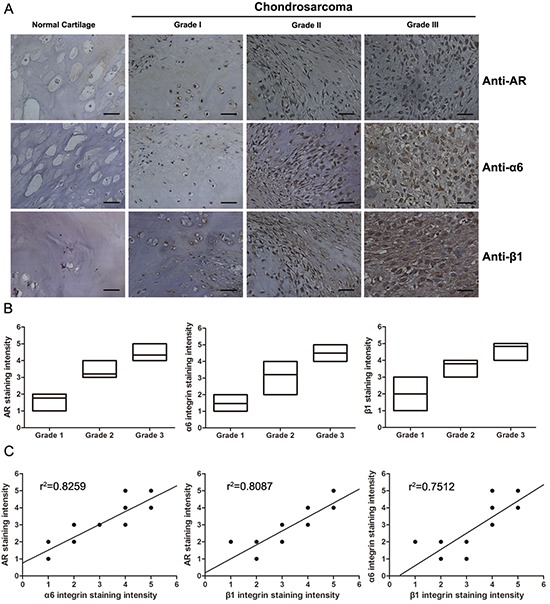
The expression levels of AR and α6β1 integrin are positively correlated with histopathological grade in human chondrosarcoma tissues **A.** IHC for AR and α6β1 integrin in representative samples of normal cartilage and different grades of chondrosarcoma tissue (grade I-III). Positive (brown) and negative (purple counterstain) staining can be readily seen in images captured with 400x magnification. Scale bars, 50 μm. **B.** Box plot comparing the expression levels of AR, α6 integrin and β1 integrin in each the histological grades of chondrosarcoma. Box plots, with the horizontal lines representing the median; the top and bottom of the boxes representing the largest and smallest observations. **C.** A scatter plot showing the correlations among AR, α6 integrin, and β1 integrin in tissue microarray observed through different grades of chondrosarcoma. The line is the best fit regression line to the points. (*P* < 0.001 for all, Pearson's correlation).

To determine the clinical significance of AR and α6β1 integrin in patients with chondrosarcoma, we utilized a tissue microarray for evaluation by IHC to compare the expression of AR, integrin α6, and integrin β1 in normal cartilage and different grades of chondrosarcoma. Representative examples of IHC staining for AR, integrin α6, and integrin β1 in normal cartilage and chondrosarcoma tissues with different grades are shown in Fig. 6A. The expression of AR, integrin α6, and integrin β1 had significantly increased with tumor progression (Fig. 6B). In addition, the Pearson's correlation showed significantly positive correlations existed between AR expression and integrin α6 (*r*^2^ = 0.8259, *P* < 0.0001), AR expression and integrin β1 (*r*^2^ = 0.8087, *P* < 0.0001), and integrin α6 and integrin β1 (*r*^2^ = 0.7512, *P* < 0.0001) (Fig. 6C). Taken together, our data indicate that elevated AR expression is associated with increased levels of α6β1 integrin and high histological grade of chondrosarcoma.

## DISCUSSION

AR is a member of the EGF family, and its increased expression has been reported in many cancers, including colorectal cancer [6, 7], breast cancer [25, 26], ovarian cancer [12, 27], pancreatic cancer [9, 28], lung cancer [8, 29], liver cancer [14, 30], oral cancer [13]. AR has been considered as a new secreted marker for exhibiting increased potential of cell invasion in cancer [31, 32]. Several factors implicated in the induction of AR expression have been demonstrated to promote cell migration and tumor metastasis. For example, the transcription factor, HOXB9, is overexpressed in breast cancer cells inducing the expression of AR, resulting in increased cell motility [33]. Conversely, Monad, a component of R2TP/prefoldin-like complex, has been shown to inhibit breast cancer cell invasion by degrading AR mRNA [32]. Furthermore, a previous study has shown that anterior gradient homolog 2 (AGR2) is associated with increased tumor metastasis [34]. More recent results, moreover, indicate that AGR2 is able to induce AR expression [35]. Another study also found that mitochondrial dysfunction has high AR expression in hepatoma cells, leading to the facilitation of cell migration [36].

Generation of the soluble forms of AR is essentially mediated by the proteolytic activity of the transmembrane proteinase ADAM-17 (a desintegrin and metalloproteinase-17) also known as tumor-necrosis factor-alpha converting enzyme (TACE) [3]. The ADAM-17/TACE have been found to be key players for the regulation of cell migration and invasion in cancer [37–39]. Conversely, inhibition of ADAM-17 can suppress cell migration and invasion [40, 41].

In lung cancer, bone is a frequent target of metastasis. A previously study discovered that AR is able to activate expression of parathyroid hormone–related protein (PTHrP) that is a causative factor contributing to osteolytic metastases [42]. With regard to the roles of AR in the relationship between tumor cells and nonneoplastic cells in the cancer microenvironment, another study also found that tumor-associated dendritic cells is able to secrete high amounts of AR, which is responsible for promoting lung cancer growth, migration, invasion, and epithelial-to-mesenchymal transition. Moreover, a markedly elevated level of AR in lung cancer patients' serum is also higher than those of healthy donors. Neutralization of AR by specific Abs significantly decreases the incidence of cancer development in mice [29]. These results suggest that anti-AR is an attractive strategy to target invasive cancer.

Due the observation that AR is highly expressed in a variety of malignancies, insights into the mechanisms related to the anti-tumor activity of AR targeted therapy might help improve chondrosarcoma therapy. In chondrosarcoma, the novel therapeutic strategies targeting AR signaling by monoclonal antibodies, soluble receptor protein, multikinase inhibitors, and RNA interference could serve as therapeutic agents for clinical application in humans. More recently, a study further demonstrated that human epidermal growth factor receptor 2 (HER2) is the only marker suited for patient selection for the trastuzumab plus pertuzumab-based regimen in HER2-positive metastatic breast cancer [43]. Therefore, high AR expression and its related receptors identify a subgroup of patients who have a high probability of responding to EGFR inhibition. It is necessary to establish whether or not expression of AR and its receptor can be used for tailoring treatment or for selecting patients for novel treatment strategies.

Previous studies have indicated that integrins are implicated in cellular migrations in chondrosarcoma [44–46]. Notably, EGF and AR have been shown to increase cell migration and invasion by enhancing integrin expression [15–17, 47, 48]. However, the detailed mechanism as to how AR-induced cell migration in chondrosarcoma remains unclear. Here, we have explored the signaling mechanism of AR in the regulation of α6β1 integrin expression in human chondrosarcoma cells. Collectively, these findings would provide a better understanding of the mechanisms underlying AR pathogenesis and can utilize this knowledge translationally for novel treatment strategies for chondrosarcoma.

## METHODS

### Cell culture

Human chondrosarcoma cell line (JJ012) was kindly provided by the laboratory of Dr Sean P Scully (University of Miami School of Medicine, Miami, FL). The human chondrosarcoma cell line (SW1353) was obtained from the American Type Culture Collection. Cells were cultured in Dulbecco's modified Eagle's medium (DMEM)/α-MEM supplemented with 10% fetal bovine serum and 100 units/ml penicillin/streptomycin at 37°C in a humidified chamber in 5% CO_2_.

### Cell migration assay

Cell migration was determined by using Transwell culture inserts (8-μm pore size; Costar, NY) according to the manufacturer's instructions. In brief, cells (1.5 × 10^4^ cells) suspended in 200 μl of serum-free medium were seeded onto the upper chamber and 300 μl of the same medium containing varying concentrations of AR (R&D Systems, Minneapolis, MN) was placed in the lower chamber. After overnight incubation, cells were washed with PBS, fixed with 4% paraformaldehyde, and stained with 0.5% crystal violet. Cells remaining on the upper surface of filter membrane were completely removed by wiping with a cotton swab. Migrated cells were then counted from six random fields under light microscope.

### Establishment of migration-prone sublines

Subpopulations of JJ012 cells were selected according to their differential migration ability using cell culture insert system as described above. After overnight migration, cells that penetrated through pores and migrated to underside of filters were trypsinized and harvested for a second round of selection. After 10 rounds of selection, migration-prone subline was designated as JJ012 (S10). Original cells were designated as JJ012 (S0) [49].

### Enzyme-linked immunosorbent assay (ELISA)

Conditioned medium was obtained from JJ012 cells grown in six-well plates. The concentrations of AR in medium were measured using Quantikine ELISA kits (R&D Systems, Minneapolis, MN) according to the manufacturer's instructions.

### Lentivirus infection and shRNA knockdown

The pLKO.1-puro-based lentiviral vectors: TRCN0000117995 (shAR#1) and TRCN0000420315 (shAR#2), and pLKO.1-shscramble were obtained from National RNAi Core Facility at Academia Sinica, Taipei, Taiwan. Recombinant lentiviruses were packaged as per manufacturer's instruction. Cultured cells were incubated with lentiviral supernatants containing 8 μg/ml polybrene for 24 h, replaced fresh medium and incubated for another 48 h. For stable cell lines, cells were selected by puromycin (5 μg/ml) [50].

### Oligonucleotide transfection

To sequester downstream signaling, Cells were transfected with MEK1 dominant-negative mutant (gifts from Dr. W. M. Fu, National Taiwan University at Taipei) or ERK2 dominant-negative mutant (gifts from Dr. M. Cobb, Southwestern Medical Center, Dallas, TX). To suppress gene expression, cells were transfected with ON-TARGET plus siRNAs targeting Ras, Raf-1, and c-Jun, and control from Dharmacon Research (Lafayette, CO). Lipofectamine 2000 (Invitrogen, Carlsbad, CA) is used to introduce oligonucleotide according to the manufacturer's recommendations.

### Quantitative real-time PCR

Total RNA was extracted using TRIzol reagent (MDBio Inc., Taipei, Taiwan) and reverse transcribed into cDNA using M-MLV reverse transcriptase, Oligo (dT), and dNTP Mix (Promega, Madison, WI) as per manufacturer's instructions. Synthesized cDNA (1 μg) was used as template for quantitative PCR (qPCR) that was conducted using KAPA SYBR FAST qPCR Kits (Kapa Biosystems, MA). Sequences for all target gene primers and probes were purchased commercially (Applied Biosystems). All primer used in qPCR were list in Supplementary Table S1. qPCR assays were carried out in triplicate using the StepOnePlus sequence detection system (Applied Biosystems). Relative gene expression was calculated using the glyceraldehyde-3-phosphate dehydrogenase (GAPDH) expression value.

### Flow cytometry

Aliquots of 1 × 10^6^ cells collected and washed once with PBS, then fixed with 70% ethanol overnight. Fixed cells were washed twice by PBS and probed with mouse monoclonal antibody specific to α6β1 integrin (1:50; Chemicon/Millipore, Billerica, MA). Cells were then washed again and incubated with fluorescein isothiocyanate isothiocyanate-conjugated goat anti-mouse secondary IgG (1:100; Leinco Tec. Inc., St. Louis, MO) for 45 min and analyzed by flow cytometry using FACS Calibur and Cell-Quest software (BD Biosciences).

### Ras pull-down assay

Cells were treated with AR in a time-dependent fashion. Activation of Ras (Ras-GTP) was detected using the Ras-binding domain of Raf-1 to pull down active Ras (Ras Activation Assay Kit from Millipore, CA, USA) according to the manufacturer's recommendations. Following separation by SDS-PAGE, proteins were transferred to membranes which were probed with an anti-RAS antibody.

### Western blotting

Cells were lysed in RIPA buffer containing protease inhibitor cocktail. Protein concentration was determined by the Pierce BCA Protein Assay Kit (Thermo Fisher Scientific Inc., Waltham, MA). Equal amount of total protein was resolved by SDS-PAGE and transferred to PVDF membranes (Millipore, Bedford, MA). Membranes were blocked with 5% bovine serum albumin and then probed with primary antibodies. After washes in tris-buffered saline with 0.05% Tween 20, membranes were subsequently incubated with appropriate peroxidase-coupled secondary antibodies. Membranes were then washed and bound antibodies were visualized using ECL reagents (PerkinElmer, MA) and autoradiography.

### Chromatin immunoprecipitation (ChIP) assay

ChIP analysis was performed as described previously [51]. Briefly, cells were fixed with 1% formaldehyde, washed, and lysed. Cell lysates were sonicated to shear DNA into smaller fragments. Protein-DNA complexes were precipitated with anti-c-Jun antibody. After reverse cross-link of protein-DNA complexs, free DNA was then extracted with phenol-chloroform. Immunoprecipitated DNA was amplified by PCR using the following primers: primers 5′-AAGCGCTCCATAAACACCTG-3′ and 5′-AATTGTGGTTGCCGAGTAGC-3′ utilized to amplify across α6 integrin promoter region; primers 5′-ACGCAACTCACCAGGTTTTC-3′ and 5′-CTAGGAGGAGGCGGAGGAT-3′ utilized to amplify across β1 integrin promoter region. PCR products were resolved by 1.5% agarose gel electrophoresis and visualized by UV light.

### Luciferase reporter assay

Cells were co-transfected with luciferase reporter gene constructs and β-galactosidase using Lipofectamine 2000 (Invitrogen) as per manufacturer's instructions. At 24 h transfection, the cells were exposed to AR for 24 h or pre-treated with inhibitors for 30 min, followed by treatment with AR for 24 h. Luciferase activity was determined using the luciferase assay kit (Promega, Madison, MA).

### *In vivo* metastasis model

Four-week-old male nude mice were used and randomly assigned to two groups (eight mice each group). Cancer cells (JJ012 (S10)/Luc or JJ012 (S10)/shAR-Luc) were resuspended in 100 μl of serum-free DMEM/α-MEM and subcutaneously injected (5 × 10^6^ cell per mouse) into the lateral tail vein of mice. Lung metastasis was monitored using an *in vivo* imaging system (Xenogen IVIS imaging system). At Day 60, mice were sacrificed by overdose with anesthetic. The lungs were then fixed in 10% formalin, embedded in paraffin and subsequently processed for IHC. All mice were handled in accordance with the Animal Care and Use Guidelines of the China Medical University (Taichung, Taiwan) under a protocol approved by the Institutional Animal Care and Use Committee.

### Immunohistochemistry (IHC)

The human chondrosarcoma tissue array was purchased from Biomax (Rockville, MD, USA; 6 cases for normal cartilage, 24 cases for grade I chondrosarcoma, 9 cases for grade II chondrosarcoma, and 15 cases for grade III chondrosarcoma). Fixed and paraffin-embedded tissues were deparaffinized with xylene, and rehydrated through a graded series of alcohols to water. Endogenous peroxidase activity was blocked with 3% hydrogen peroxide Heat-induced antigen retrieval was carried out for all sections in 0.01 M sodium citrate buffer, pH 6 at 95°C for 20 min. Human AR, integrin α6, and integrin β1 antibodies were applied at a dilution of 1:200 and incubated at 4°C overnight. Bound antibodies were detected by NovoLink Polymer Detection System (Leica Microsystems, Newcastle, UK) and visualized with the diaminobenzidine reaction. The sections were counterstained with hematoxylin. IHC results were scored by taking into account the percentage of positive detection and intensity of the staining.

### Statistics

All data are presented as mean ± SEM. Statistical comparison of two groups was performed using the Student's *t*-test. Statistical comparisons of more than two groups were performed using one-way analysis of variance with Bonferroni's *post hoc* test. In all cases, *P* < 0.05 was considered significant.

## SUPPLEMENTAL TABLE


