# Correction: Amphiregulin enhances alpha6beta1 integrin expression and cell motility in human chondrosarcoma cells through Ras/Raf/MEK/ERK/AP-1 pathway

**DOI:** 10.18632/oncotarget.16998

**Published:** 2017-04-10

**Authors:** Jui-Chieh Chen, Yu-Ju Chen, Chih-Yang Lin, Yi-Chin Fong, Chin-Jung Hsu, Chun- Hao Tsai, Jen-Liang Su, Chih-Hsin Tang

**Present**: Due to an error during figure assembly, Figure 4C mistakenly contains the same image as Figure 3D.

Correct: The proper Figure 4C is shown below. The authors sincerely apologize for this oversight. The conclusions of the paper remain unchanged.

Original article: Oncotarget. 2015; 6:11434-46. doi: 10.18632/oncotarget.3397

**Figure 4:**
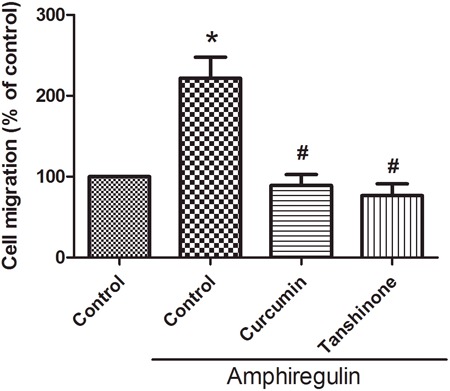
Activation of c-Jun is required for AR-induced cell migration and up-regulation of integrin α6β1 **C.** Cells were pretreated with curcumin (10 μM) or tanshinone (10 μM) for 30 min followed by stimulation with AR (50 ng/ml) for 24 h. The *in vitro* migration was measured by Transwell assay. Results are expressed as mean ± SEM. *P < 0.05 compared with control; #*P *< 0.05 compared with AR-treated group.

