# Supplementary material for: Amphiregulin enhances alpha6beta1 integrin expression and cell motility in human chondrosarcoma cells through Ras/Raf/MEK/ERK/AP-1 pathway
Source: Oncotarget. 2015 Mar 18;6(13):11434–46. doi: 10.18632/oncotarget.3397 (PMC4484467; doi:10.18632/oncotarget.3397)
Supplement: Supplementary file 1 [file oncotarget-06-11434-s001.pdf]

SUPPLEMENTAL TABLE

Supplementary Table S1. RT-PCR primer sequence.

αv(F)	5′-ACTGGGAGCACAAGGAGAACC-3′
αv(R)	5′-CCGCTTAGTGATGAGATGGTC-3′
α5(F)	5′-CAGACCCTGCTCATCCAGAAT-3′
α5(R)	5′-GGCATTCTTGTCACCCAGGTAC-3′
α6(F)	5′-GAGGAATATTCCAAACTGAACTAC-3′
α6(R)	5′-GGAATGCTGTCATCGTACCTAGAG-3′
β1(F)	5′-AGGATTACTTCGGACTTCAGA-3′
β1(R)	5′-CTTTGGCATTCACATTCA-3′
β3(F)	5′-TGCTCATTGGCCTTGCCGCCCTGC-3′
β3(R)	5′-ACTATTCGTCAGTAGGAGTCTAGT-3′
β5(F)	5′-CACAGCACCGAGATACCAGA-3′
β5(R)	5′-CTGTCTGTGAGAGGCAGCAG-3′
